# An Animal-Assisted Education Intervention with Dogs to Promote Emotion Comprehension in Primary School Children—The Federico II Model of Healthcare Zooanthropology

**DOI:** 10.3390/ani11061504

**Published:** 2021-05-22

**Authors:** Cristiano Scandurra, Antonio Santaniello, Serena Cristiano, Fabrizio Mezza, Susanne Garzillo, Rosa Pizzo, Lucia Francesca Menna, Vincenzo Bochicchio

**Affiliations:** 1 Department of Neuroscience, Reproductive Sciences, and Dentistry, University of Naples Federico II, 80131 Naples, Italy; cristiano.scandurra@unina.it (C.S.); ros.pizzo@virgilio.it (R.P.); 2 Department of Veterinary Medicine and Animal Productions, University of Naples Federico II, 80134 Naples, Italy; antonio.santaniello2@unina.it (A.S.); susannegarzillo@gmail.com (S.G.); 3 SInAPSi Center, University of Naples Federico II, 80133 Naples, Italy; serenella25@live.com (S.C.); fabrizio.mezza92@gmail.com (F.M.); 4 Department of Humanistic Studies, University of Calabria, 87036 Rende, Italy; vincenzo.bochicchio@unical.it

**Keywords:** emotional development, animal-assisted intervention, inter-specific relationship, human–animal interaction, school, dogs, one health

## Abstract

**Simple Summary:**

Children’s understanding of the nature, causes, and regulation of emotions represents a crucial developmental competence, as it improves the quality of peer interactions and increases educational success. This study presents an animal-assisted education intervention model with dogs to promote emotion comprehension in a group of children aged 6–7 years. Children who benefitted from the intervention improved their emotion comprehension compared to children who did not benefit from it. This improvement may be due to the beneficial role of relationships with companion animals, as they seem to positively affect social and emotional development in children, as well as enhancing their social competence, emotion regulation, and empathy. Further psychological processes which may have influenced the positive outcomes achieved are the group dynamics and role model offered by the relationship between the dog and the zootherapist veterinarian.

**Abstract:**

Emotion comprehension (EC) is a crucial competence for children, as it determines the quality of peer interactions. This study assessed the efficacy of an animal-assisted education (AAE) intervention with dogs based on the Federico II Model of Healthcare Zooanthropology (FMHZ) to promote EC in a group of primary school children. One hundred and four children (48 females) aged 6–7 years took part in the study, of whom 63 participated in the AAE intervention (i.e., experimental group) and 41 did not (i.e., control group). The intervention was deployed in a school setting through a group format and consisted of five bimonthly sessions. EC was assessed pre- and post-intervention, and at a 3-month follow-up. Student’s t-test and mixed-model ANOVA were performed to analyze the effect of the intervention on EC. EC significantly improved in children of the experimental group compared to the control group. Significant time effects from pre- to post-intervention, post-intervention to follow-up, and pre-intervention to follow-up assessment were found in the experimental group only. AAE based on FMHZ was effective in improving EC in children.

## 1. Introduction

The benefits of human–animal interaction (HAI) on children’s socio-emotional development and wellbeing are documented in a growing body of evidence [1]. Positive interactions with companion animals, whether they occur in family, recreational, educational or clinical contexts, can improve emotion regulation in children, enhancing their empathic skills and helping them mitigate stressful emotions [2]. HAI research has been informed by multiple theoretical frameworks arguing the role of companion animals in influencing child development and human psychophysical wellbeing. For instance, the biophilia hypothesis [3] postulated an innate affinity of humans of all ages to the natural environment and to the living beings around them. Ecological systems theory [4], in postulating that human interactions with the environment would significantly influence their development, suggested that the presence of companion animals in a child’s environment has significant implications for their developmental trajectory. Theories such as the social support theory [5] and the attachment theory [6,7], that were primarily focused on interpersonal relationships and parent–child bonds, have been applied to HAI research, providing a basis to explain human–animal relationships and the mechanism through which HAI positively affects child health and development.

In light of the growing evidence of the beneficial effects of HAI on children’s social and emotional development [1,8,9,10], the inclusion of animals in educational contexts has gained increasing popularity in recent decades [2]. Animal-assisted education (AAE) interventions are widely used to promote the development of emotional, interpersonal, and cognitive skills in both typically developing children and children with special educational needs (e.g., those with autism spectrum disorder) [11]. Positive outcomes of AAE interventions for children include better attitudes toward school attendance and learning, reduced stress, development of positive emotions, empathy, better task performance, improved classroom cohesion, prosocial behaviors, and decreased aggression [11,12].

Among the socio-emotional developmental skills which AAE interventions are able to promote in children [13], in this study we addressed emotion comprehension (EC). EC may be defined as children’s understanding of the nature, causes, and regulation of emotions, as well as the children’s ability to identify, predict, and explain emotions for themselves and to others [14]. According to the developmental model by Pons et al. [15], EC occurs between 3 and 11 years, and despite the existence of a certain amount of individual variability, children with a typical developmental profile progress along three main levels of EC. First, at the “external” level, children aged 3–5 years recognize facial expressions (e.g., sadness, happiness, fear, and anger) and understand the impact of situational factors on emotions, as well as the role of desires in emotions. Second, at the “mental” level, children aged 6–7 years understand the role of beliefs in emotions, the impact of memory on emotions, and the distinction between outwardly expressed and privately felt emotions. At the third “reflective” level, children aged 8–11 years understand how moral considerations may affect emotions, recognize that emotions may be regulated by means of cognitive control strategies, and are aware of concurrent mixed feelings. Each transition from one level to another represents an increase in the child’s ability to understand the effect of internal states on emotional experience. EC is crucial for children as it may determine the quality of interactions with peers, with a positive impact on educational success; by contrast, emotional dysregulation is often associated with aggressive attitudes, influencing the risk of school dropout [16]. For these reasons, many scholars have highlighted the need to promote the development of emotional competence and awareness in children, as it may be a crucial protective factor against negative health and educational outcomes (e.g., emotion dysregulation, school dropout) in educational contexts [17,18,19].

This study presents an AAE with dogs to promote EC in a group of primary school children based on the Federico II Model of Healthcare Zooanthropology (FMHZ) [20,21]. Before describing the method we used and the results of the intervention, in the following section we describe the applied theoretical model.

### The Federico II Model of Healthcare Zooanthropology

The FMHZ [20] is rooted in complexity theory [22], identifying the animal-assisted interventions (AAIs) setting as a dynamic and complex system encompassing many interdependent variables, the relationships among which might be nonlinear [21]. By assuming a holistic and systemic perspective, the FMHZ emphasizes the relational nature of the dynamics taking place within the AAI setting.

The FMHZ also refers to the concept of animal reference, which is the basis of the zooanthropology discipline [20,23,24]. From this perspective, the animal is considered a hetero-specific subject that actively contributes to the development of human relationships. Thus, the animal is considered an active part of the clinical and/or educational process and as a catalyst of the transformative processes activated within the setting. To this end, the FMHZ provides clinical and educational interventions based on human–animal interactions, which in clinical or educational settings are named inter-specific relationships (ISRs).

The FMHZ adopts an interdisciplinary approach by using an inter-specific team comprising a zootherapist veterinarian and a psychologist (as an inter-specific relationship expert), both of whom have a specific training in human–animal interaction, and a dog. The zootherapist veterinarian is the dog handler and participates in all AAI meetings as the guarantor of the safety of the ISR within the intervention context. Furthermore, the zootherapist veterinarian has the task of controlling health and behavioral aspects related to the user’s interaction with the animal. The psychologist and the zootherapist work together to organize psychological activities with the animal. Indeed, the psychologist is responsible for the human emotional dynamics occurring in the classrooms, structures the zootherapeutic intervention, maintains customer relations, and defines the objectives of the intervention and, where necessary, the assessment tools. The working team is considered a therapeutic system in which every member is aware and responsible for their own functions, resources, and skills [20].

The FMHZ involves dogs, one of the main species included in AAIs [25]. Dogs are chosen for their natural attitudes of openness, affection, curiosity, and sociability, as they are often considered a facilitator within social relationships. Indeed, largely based on non-verbal language, the ISR with a dog can facilitate the free circulation of emotions, favoring an atmosphere of respect and acceptance. Indeed, since animals seem more straightforward in their emotional displays, children find it easier to read them and are more prone to develop confidence in them as a result [8,26]. Over the course of their history of coevolution with humans, dogs have developed highly sophisticated communicative skills which allow them to tune into human emotional states [27] by reading the non-verbal language, proxemics, and behaviors of the human being [28,29]. Through contact with dogs, people involved in the intervention are able to reflect on themselves thanks to a visual contact, which reinforces the intimacy and results in the development of a communicative code of gestures and sounds that are primarily based on non-verbal language [30]. Indeed, observing gestures and behavioral strategies of the inter-specific partner may elicit in the human a mirroring process, which may promote reflection and a better understanding of one’s own experiences, attitudes, resources, and limits. To this end, the psychologist would allow the human to reflect on mental and emotional content that emerges from the relationship, encouraging self-reflection and awareness [20].

This study aimed to assess the efficacy of an AAE with dogs to promote age-appropriate EC abilities in a group of children aged 6–7 years. The principal hypothesis was that an AAE intervention based on the FMHZ can improve children’s EC level.

## 2. Materials and Methods

### 2.1. Participants and Procedures

One hundred and four children (56 males and 48 females) with an average age of 6.55 (standard deviation [SD] = 0.50) years who attend the second grade of two different schools in a city in southern Italy took part in the current study. Classes of children participated in the current intervention on the basis of teachers’ and schools’ availability, thus representing a convenience sample. Specifically, 3 classes belonging to one of the two schools were assigned to the experimental group (*n* = 63, of whom 35 were male and 28 female) and participated in an AAE intervention, while the 2 classes of the other school were assigned to the control group (*n* = 41, of whom 21 were male and 20 female) and did not participate in the intervention. Children assigned to the control group participated in their usual educational activities. However, teachers were asked to concentrate such activities on EC.

The intervention was deployed in a group setting, as the group may help participants build new meanings about proposed contents, facilitating interactive exchanges, and expanding their points of view on the topic of intervention (in this case, emotions). Furthermore, the active group can facilitate the emergence of new reflections and the recognition of new dialogic and relational skills against other points of view [31,32,33].

All data were collected in accordance with the Italian Law on Privacy and Data Protection 196/2003 and the European Union Regulation 2016/679 “General Data Protection Regulation” and became property of the Department of Veterinary Medicine and Animal Productions of the University of Naples Federico II. They were stored in a database accessible only to the principal investigator. The study was conducted according to the guidelines of the Declaration of Helsinki and approved by the Institutional Review of the University of Naples Federico II (protocol code: 188/19; approved 6 January 2019). Informed consent for each child was obtained from parents before the intervention. Parents were invited to participate in a public event during which they were informed about the objectives, measures, times, and activities related to the project. Furthermore, we asked parents if their children had allergies or were scared of dogs. No children involved met these conditions.

### 2.2. The Dog

Within the current intervention, a dog was considered as particularly functional to the children, as children tend to develop trustful relationships with companion animals [34,35,36], turning to them for social support in emotionally stressful situations [28], and gaining various benefits from interactions with them in terms of wellbeing, health, resilience, and EC [11,37,38,39].

The dog involved in this study was chosen following a canine Monash personality test, and having achieved a good score based on extroversion, focus motivation, and friendliness [40]. This test was useful to highlight the dog’s high relational ability [41,42,43,44].

The dog, named Leo, was a male Cavalier King Charles that was aged 16 months at the time of the experiment. The dog was chosen for its ability to relate in a composed and tender manner with people, as well as its inclination to seek contact with humans through requests for care, play, and affection. Both the dog and its veterinarian handler were trained in ISR with a specific focus on children, having participated in an educational program which was held at a center in southern Italy that follows the FMHZ guidelines.

In addition, before having access to the classroom and after the end of each AAE session, disinfectant wipes (i.e., chlorhexidine, TRIS-EDTA, zinc gluconate, and glycerine) were used to clean the coat, paws, and tail of the dog to avoid the transmission of zoonotic agents (e.g., bacteria, fungi, parasitic elements) [24,45,46,47,48,49].

### 2.3. The Intervention

The intervention consisted of five bimonthly group sessions, each lasting 1 h, that took place between February and April 2019 and were preceded by an introductory session in January 2019.

The intervention was developed with the aim of enhancing EC competency in children. To this end, on the basis of the theoretical framework by Pons et al. [15], four primary emotions (i.e., joy, fear, sadness, and anger) were chosen. Indeed, the authors advised against evaluating more complex emotions (e.g., shame) as they might be difficult for younger children to recognize. Specifically, the intervention was structured to facilitate the recognition of the dog’s emotional experiences by the children through its presence and/or reading the situation stimuli, the latter being both dog behaviors acted at that time and stories told in which the protagonist was a dog.

Each meeting consisted of three periods: (1) the children were initially free to play with the dog for approximately 10 min; (2) the psychologist then introduced the topic of the session in the presence of the zootherapist veterinarian and the dog; (3) and finally, the psychologist conducted a group discussion with the children in the presence of the zootherapist veterinarian and the dog.

The working methodology was managed according to ISO 9001-2015 (Cert.n.317jSGQ10). The topics of the sessions were as follows.

#### 2.3.1. First Session: “Which Dog Do You Want to Be?”

The first meeting was aimed at introducing the inter-specific team to the children. Pictures and video recordings of dogs were shown to the children, with the distinction between the human and the other animal species pointed out. While describing the differences in morphology and in communication, children were asked to empathize with dogs. With the help of their teachers, they also created dog ears and tails.

#### 2.3.2. Second Session: “As It Moves the Joy”

The primary emotion addressed in this session was joy. With the aim of engaging both the children and the dog, we proposed play comprising two games. The whole group of children was involved in the “Chinese Whispers” game, during which each child had to whisper a message to the ear of the next child along a line, until the last player announced the message to the entire group. In the meantime, children had to pass a ball to the next child without dropping it, even involving the dog. This game was intended to create a playful relationship between the dog and the group class rather than between the dog and a single child.

#### 2.3.3. Third Session: “Brrrr…What a Fear!”

The primary emotion treated during the third session was fear. During this session, we changed the setting, darkening it as much as possible to simulate a mysterious environment (e.g., a woodland). Successively, children were asked to interpret an object or an animal, keeping their eyes closed, so that they could not see the dog but had to perceive it with other senses. A fairy tale in which the dog was the protagonist (as a wolf) was told by the zootherapist, walking the dog among children until there was a direct contact via food placed on the children’s shoes, arms, or back.

#### 2.3.4. Forth Session: “The Silhouette of Sadness”

The primary emotion treated in the fourth session was sadness. In this session, a game tool named “otherness’s dice” was used. Six different images were depicted on a die (3 regarding a child and 3 regarding Leo the dog), each representing a different nuance of sadness. Children had to throw the die into the center of the group, and the children were then asked to mimic the emotional expression of both the child and Leo by using the tail or ears built in the first session.

#### 2.3.5. Fifth Session: “Mr. Anger”

The primary emotion of the fifth session was anger. During this session, photo and video materials relating to dogs were used (e.g., a short video about Leo interacting with his parents when he was a puppy was shown). These materials exhibited behaviors of anger aimed at containing and educating the puppies. In this way, the children were able to observe the dog’s calming signals and we were able to highlight the importance of recognizing and expressing these emotions consciously.

#### 2.3.6. Sixth Session: “Wow…Now What Happens?”

The last session did not take place in the classroom but in another larger area of the school. In this meeting, another four zootherapists who were part of the working group brought their dogs, allowing the children to interact freely with them and showing them the communication and behaviors of dogs through the expression of their emotions. At the end of this session, a conclusive group reflection with the children on the whole experience was carried out.

### 2.4. Assessment

The Test of Emotion Comprehension (TEC) [50,51] was administered pre-intervention, post-intervention, and at a 3-month follow-up to both the experimental and control groups. The TEC is a measure used to assess the understanding of the nature, causes, and regulation of emotions in children aged 3–11 years. Specifically, the TEC assesses 9 components of emotion comprehension: recognition, cause, desire, belief, reminder, regulation, hiding, mixed, morality. Such components refer to three levels of emotion comprehension: external, mental, and reflective. The mental level of EC is typical of children aged 6–7 years, so in the current study we administered only cards about components of this scale.

The TEC administration procedure consisted of showing each child 23 images in which the protagonist has a blank face, and the experimenter tells a story relating to the single images. Subsequently, four faces were shown with different emotional expressions and the child was asked to indicate the emotion most pertinent to the story. Depending on the participant’s gender, a corresponding version of the book with either female or male protagonists was presented.

A total score was obtained by assigning 1 point for each component answered correctly, with an overall score of emotion understanding ranging from 0 to 9. In the current study, we normalized and scaled scores to a range of 0–1. The assessment was performed by two independent judges. Previous studies have reported high test–retest reliability and good concurrent validity of the measure [52].

### 2.5. Statistical Analysis

Statistical analysis was performed using IBM SPSS Statistics 26 and we considered results of statistical tests to be significant at *p* < 0.05. Before proceeding with data analysis, the presence of outliers was explored following the recommendations by Tabachnick and Fidell [53], i.e., by verifying the presence of standardized scores greater than 3.29 or less than –3.29. No outliers were found. Furthermore, there were no missing values since the investigator personally administered the TEC to each child individually.

We tested differences in TEC scores between the experimental and control groups using a Student’s t-test. The effect size was calculated using Cohen’s *d* (*d*; small effect = 0.01, medium effect = 0.06 and large effect = 0.14) [54].

Then, a 3 (Time) × 2 (Group) mixed-model analysis of variance (ANOVA) with Bonferroni adjustments was performed to analyze the effect of the intervention on the TEC (dependent variable). “Time” refers to the three measurement times (i.e., pre-intervention, post-intervention, and 3-month follow-up assessments), while “Group” refers to the experimental and control groups. In case of violation of the sphericity assumption, Greenhouse–Gressier statistics were reported. Finally, with the aim of controlling for the potential effects on TEC of participants’ age, gender, and belonging to a specific class, these variables were included as covariates in the ANOVA. The effect size was calculated using Cohen’s *η*^2^ (*η*^2^; small effect = 0.01, medium effect = 0.06 and large effect = 0.14) [54].

## 3. Results

As shown in Table 1, the control and experimental groups were similar at baseline, but differed significantly at both the post-intervention and 3-month follow-up assessments, with the experimental group showing higher TEC scores.

The mixed-model ANOVA revealed that the main effect for Group was significant (F (1102) = 5.07, *p* = 0.03, *η*^2^ = 0.05), indicating that there was a significant difference in TEC scores between the experimental and control groups, although the effect size was small. A significant main effect for Time was obtained with a large effect size (F (1102) = 18.13, *p* < 0.001, *η*^2^ = 0.15), indicating that the TEC scores significantly differed across the three times of intervention.

**Figure 1:**
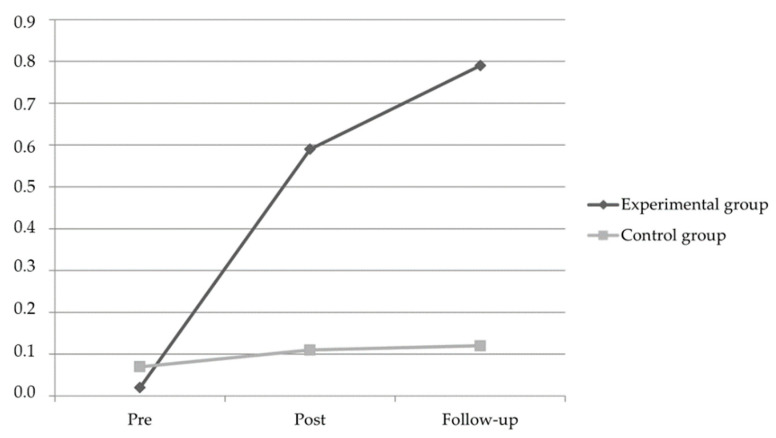
Changes in Test of Emotion Comprehension (TEC) scores in the experimental and control groups at pre- and post-intervention, and at the 3-month follow-up assessment.

Additionally, a significant Time × Group interaction was found with a large effect size (F (1226) = 13.37, *p* < 0.001, *η*^2^ = 0.12). Indeed, post hoc tests using Bonferroni correction showed significant time effects from pre- to post-intervention, post-intervention to follow-up, and pre-intervention to follow-up only in the experimental group, while in the control group there were no statistically significant differences among times periods. Pairwise comparisons in both the experimental and control group and for all the times of measurements are shown in Table 2 and Figure 1.

Finally, class (F (1102) = 0.24, *p* = 0.62), age (F (1102) = 0.09, *p* = 0.75) and gender (F (1102) = 0.01, *p* = 0.93) were not statistically significant covariates, indicating that these factors did not significantly impact the changes detected.

## 4. Discussion

In this study, we evaluated the efficacy of an AAE intervention with a dog based on the FMHZ principles to enhance age-appropriate EC abilities in a group of primary school children. Our results show that this intervention was effective in improving EC in children, as the assessed mental level of EC increased significantly in the experimental group compared to the control group. To the best of our knowledge, this is the first study that has focused on a training program aimed at enhancing EC through the involvement of a dog acting as the catalyst and measured using the TEC.

Previous studies have similarly demonstrated that involving pets in classrooms can enhance children’s social interactions [55,56]. However, most studies have been focused on AAE in special education classrooms (i.e., with children with autism spectrum disorder, attention-deficit/hyperactivity disorder, or emotional and behavioral disorders) [56,57,58], while few have addressed general education classrooms [56,59,60,61,62]. To this end, previous studies examined the impact of dogs on the classroom’s dynamics, showing that the presence of an animal was directly related to increased social cohesion and decreased aggression among children aged 6–10-years [60,61], as well as increased cognitive task performance among children aged 3–5-years [63,64].

In our study, the experimental group exhibited a greater improvement in the mental level of the EC compared to the control group. The mental level of EC is related to seeing things with more perspective, meaning that children learn that other people can experience different emotions regarding the same object or situation, depending on their desires or beliefs about it (e.g., being aware of danger or not), and that a person’s outward emotional expression can deviate from the internal emotional experienced [15]. Our study suggests that an AAE intervention structured using FMHZ principles might improve these abilities. This is line with the previous studies that reported the beneficial role of relationships with companion animals in affecting social and emotional development in children, as well as in enhancing their social competence, emotion regulation, and empathy [1,9,10]. These beneficial effects seem to be due to the ISR rather than the mere presence of an animal [65]. Indeed, the FMHZ gives particular attention to the ISR as a facilitator to have access to a play area. To this end, recognizing the animal’s otherness may have allowed the children to explore their own and others’ emotions without perceiving themselves to be invaded or judged, in such a way that a free circulation of emotions could happen. Furthermore, the presence of the animal (mediated by the handler) may have created a context promoting the understanding of one’s own resources and limits, in a climate of reciprocal respect and acceptance [20]. According to the animal reference principle [66], the more the dogs are recognized for their otherness, the greater the chances are of tuning into them, reading their behaviors in terms of emotional experiences through their non-verbal language, and benefitting from the process.

Moreover, our results are in line with the idea that interactions with animals may provide children with increased opportunities to practice perspective-taking, leading to earlier or more sophisticated theory-of-mind development [67,68]. A significant body of evidence has revealed positive correlations between theory-of-mind and EC competencies [19,69,70], with recent studies showing that children’s EC precedes and influences their acquisition of theory-of-mind skills [16].

Furthermore, other psychological processes that may have influenced the positive outcomes achieved include the attention given to the relationship between all members involved, and the role model offered by the relationship between the dog and the zootherapist veterinarian. With regard to the first point, the relationship between all members involved was fostered by the introductory period of each meeting, when the children were left free to play with the dog in the presence of the psychologist and the veterinarian. This warm-up phase was crucial to help both the children and the dog to become familiar and recognize each other through the sessions, and this promoted a playful and comfortable atmosphere in which the subsequent goal-oriented phases could take place. Across the sessions, a trustworthy and safe relationship was developed between the children, the dog, and the working team, helping to create an accepting, nonjudgmental, empathetic, and respectful setting which enabled the children to explore their emotions through the games and activities. As confidence increased, it was possible to move on to increasingly demanding activities, corresponding to increasingly challenging emotions. By observing, reading, and miming the dog’s emotional displays and behavior, the children were encouraged to understanding the nuances and displays of the main emotions—joy, fear, sadness, and anger—and to appreciate their social functions in communication and interactions.

With regard to the relationship between the dog and the zootherapist veterinarian, dogs appear to be particularly sensitive to the relationship with their owners, as it gives them a sense of security and confidence [23,71,72]. The mutual trust and empathy within the relationship between the dog and the zootherapist veterinarian may have functioned as a basis for the emotional regulation of the group. Indeed, the zootherapist’s attitude toward the dog, empathetic but simultaneously rigorous, may be viewed as a behavioral model for children in their mutual relationships and in their own relationship with the dog [21].

The results obtained in this study should be read in light of some limitations. The first concerns the sample. Indeed, the sample size was relatively small and included only children aged 6–7 years. This means that, according to the model by Pons et al. [15], we worked only on the “mental” level of EC. Furthermore, being a convenience sample recruited on the basis of the teachers’ and schools’ availability, this study should be considered a pilot trial. Future studies should replicate this kind of intervention with a larger number of participants, encompassing both younger and older children to explore potential differences in the outcome in terms of age and EC level, as well as conducting an a priori power analysis on the sample size to ensure that the number of participants is sufficient to achieve adequate power. Second, we did not compare AAE interventions based on the FMHZ with AAE interventions based on other theoretical frameworks, and this did not allow us to examine whether our findings were a result of the specific intervention model or were simply a result to other general factors (e.g., the presence of the dog) or psychological ones (e.g., role playing activities, being involved in group discussions guided by the psychologist). Future studies should perform randomized controlled trials to compare FMHZ-based AAE interventions with other animal-assisted models. Third, although teachers of children assigned to the control group were asked to concentrate their usual educational activities on EC, it was not the researchers who performed such activities, and this did not guarantee the implementation of a controlled experiment. Future research should replicate this study by including a control group exposed to exactly the same conditions but without the presence of the animal. Fourth, although we postulated that specific processes may have influenced our results (e.g., relationship between all members, role model offered by the relationship between the dog and the zootherapist veterinarian), we did not assess the AAE processes, and our interpretations are only speculative. Future studies should implement a more complex assessment procedure, analyzing both outcomes and processes.

## 5. Conclusions

Our study provides insights into the emerging and rapidly expanding field of AAE, despite some limitations. Indeed, this work has demonstrated that AAE may be an effective method to promote the meal level of EC in children, and provides further evidence of the beneficial effects of AAE on emotional outcomes in children. However, the mechanisms through which the presence of an animal leads to beneficial outcomes remain to be elucidated. In addition, univocal data concerning the methodological aspects of the interventions, in particular relating to the structuring of the settings (i.e., number and length of sessions, duration of treatment, operators), are still lacking.

## Figures and Tables

**Table 1:** Comparison of mean TEC scores between the experimental and control groups at the three measurement times.

	Experimental Group (*n* = 63)	Control Group (*n* = 41)			
Time	M (SD)	M (SD)	t	95% CI	d
Pre-intervention	0.02 (0.89)	0.07 (0.94)	−0.24	−0.41, 0.32	0.05
Post-intervention	0.59 (0.97)	0.11 (0.82)	2.61 *	0.12, 0.83	0.53
Follow-up	0.79 (0.97)	0.12 (0.82)	3.66 ***	0.32, 1.02	0.74

TEC = Test of Emotion Comprehension; M = mean; SD = standard deviation; CI = confidence interval; d = Cohen’s d. * *p* < 0.05; *** *p* < 0.001.

**Table 2:** Pairwise comparisons in the experimental and control groups of TEC scores between assessment time periods from pre- to post-intervention, post-intervention to follow-up, and pre-intervention to follow-up.

	Experimental Group (n = 63)				Control Group (n = 41)			
Time	MD	SE	*p*	95%CI	MD	SE	*p*	95%CI
Pre to post	0.57	0.11	<0.001	0.30, 0.84	0.05	0.14	1.00	−0.29, 0.39
Post to follow-up	0.20	0.01	<0.001	0.20, 0.20	0.01	0.01	0.25	−0.01, 0.02
Pre to follow-up	0.77	0.11	<0.001	0.20, 1.04	0.06	0.14	1.00	−0.29, 0.40

TEC = Test of Emotion Comprehension; MD = mean difference; SE = standard error; CI = confidence interval.

## Data Availability

Anonymized data will be made available upon reasonable request to the corresponding author.
